# Emerging human pulmonary dirofilariasis in Hungary: a single center experience

**DOI:** 10.1186/s13000-024-01507-z

**Published:** 2024-06-21

**Authors:** Levente Kuthi, Tamás Zombori, László Tiszlavicz, Fanni Hegedűs, Szintia Almási, Bence Baráth, Mohammed Almakrami, Mohammad Jamal EJ, Nikolett Barta, Zsuzsanna Ujfaludi, Tibor Pankotai, Adrienn Hajdu, József Furák, Anita Sejben

**Affiliations:** 1 Department of Surgical and Molecular Pathology, Tumor Pathology Center, National Institute of Oncology, Budapest, Hungary; 2 Department of Pathology and Experimental Cancer Research, Semmelweis University, Budapest, Hungary; 3 Department of Pathology, Albert Szent-Györgyi Medical School, University of Szeged, Állomás utca 1, Szeged, H-6725 Hungary; 4 Centre of Excellence for Interdisciplinary Research, Development and Innovation, University of Szeged, Szeged, Hungary; 5 Hungarian Centre of Excellence for Molecular Medicine (HCEMM), Genome Integrity, Szeged, Hungary; 6 Competence Centre of the Life Sciences Cluster of the Centre of Excellence for Interdisciplinary Research, Development and Innovation, University of Szeged, Szeged, Hungary; 7 Medipredict, Budapest, Hungary; 8 Department of Surgery, Albert Szent-Györgyi Medical School, University of Szeged, Szeged, Hungary

**Keywords:** Coin lesion, Human pulmonary dirofilariasis, Hungary, Polymerase chain reaction

## Abstract

**Background:**

Human pulmonary dirofilariasis (HPD) is rare in Hungary, and it stems from *Dirofilaria immitis*, mainly transmitted through mosquito bites, with dogs as primary hosts. Despite its prevalence in veterinary settings, human cases are infrequent. Historically, Mediterranean countries report most HPD cases, but sporadic cases occur in temperate European regions. Radiologically, HPD often manifests in a non-specific manner, resembling pulmonary neoplasms, leading to unnecessary surgery and patient distress.

**Methods:**

This study presents a notable case series from Hungary, encompassing a 12-year period, documenting 5 instances of HPD with the aim to provide baseline estimate of occurrence for future comparison.

**Results:**

Among the patients studied, all were of middle age (median: 52 years, range: 37–69) and exhibited tumor-like lesions, primarily localized to the right lung, necessitating lobectomy or wedge resection. Histological examination consistently revealed a necrotizing granulomatous response characterized by remnants of helminths, without the presence of ovules. Furthermore, rigorous diagnostic procedures excluded other potential infectious agents through specialized staining techniques. Polymerase chain reaction analysis definitively confirmed the diagnosis of HPD in each case.

**Conclusions:**

This case series highlights HPD as a seldom zoonosis, with a probable escalation in its occurrence within temperate regions. Therefore, clinicians should maintain a heightened awareness of HPD in the differential diagnosis of pulmonary coin lesions. Early recognition and diagnosis are paramount for appropriate management and prevention of potential complications associated with this increasingly recognized infectious entity.

## Background

Pulmonary dirofilariasis, caused by *Dirofilaria immitis*, commonly known as the dog heartworm, primarily affects domestic dogs, serving as the usual definitive host. However, various mammalian species, particularly carnivores, can also become infected in endemic regions [1, 2]. The transmission of this parasitic infection primarily occurs through mosquito vectors, predominantly species such as *Culex* spp., *Aedes* spp., and *Anopheles* spp. Additionally, certain fleas, lice, and ticks may act as alternative vectors [3, 4].

Adult heartworms typically inhabit the pulmonary arteries and the right chambers of the heart in their definitive hosts, although instances of presence in the left cardiac chambers have been documented, as well [5, 6]. Female heartworms are viviparous, releasing numerous microfilaria larvae daily into the bloodstream. These larvae are ingested by blood-sucking mosquitoes, where they develop into infective larvae. Upon mosquito bite, these larvae are transmitted into the dermis of the final host, where they undergo several months of migration and maturation before reaching their final adult stage [2, 6, 7].

Via the venous circulation, these mature parasites may even reach the host’s right ventricle [1]. Adult heartworms can reach lengths of up to 25–31 cm [8]. Human hosts are infected in a similar manner to natural animal hosts; however, humans serve as accidental, dead-end hosts for dirofilariae [3]. The worms fail to reach maturity in the heart, perishing in the right ventricle instead. The resulting dead larvae embolize into the pulmonary circulation, causing minor hemorrhagic infarctions, surrounded by granulomatous reactions. This process manifests as peripheral coin lesions, detectable on chest X-rays or computed tomography (CT) scans, often mistaken for pulmonary neoplasms [9]. Although typically asymptomatic, embolization may occasionally lead to chest pain, cough, and hemoptysis [3]. Importantly, blood eosinophilia is not a consistent characteristic of the infection [10].

## Methods

All cases of human pulmonary dirofilariasis surgically resected at the University of Szeged between January 1, 2012 and December 31, 2023 (*n* = 5) were compiled for this study. Patient data, including sex, age, localization of the tumor-mimicking lesion, clinical presentation, type of surgical treatment, and available social factors, were extracted from medical records.

Regarding macro- and micromorphology, grossing data such as size and general appearance, histological features, examinations to identify infectious origin, and polymerase chain reaction (PCR) results were analyzed. Orcein staining was conducted in all cases to highlight vascular structures, thereby confirming the precise presence of helminths. The diagnosis of HPD was established in all cases through PCR analysis.

### PCR analysis

From the most representative formalin-fixed and paraffin-embed block of each case, a 10-µm-thick slide was transferred to 1.5 mL Eppendorf tubes after selection of the area of interest by an expert pathologist and deoxyribonucleic acid (DNA) was extracted by using the ReilaPrep FFPE qDNA Miniprep kit (#A2352, Promega, USA), following the manufacturer’s instructions. The final elution volume was 20 µl in pyrogen and RNAse-free water. The quality and concentration of the isolated DNA was determined by using Qubit 4 fluorometer (Thermo Fisher Scientific), according to the manufacturer instruction. Specific gene and genomic regions of *Dirofilaria immitis* and *Homo sapiens* were amplified by Taq polymerase in a final volume of 20 µL in 0.5 mL tubes containing 0.3 µM of each primer and 50 ng of DNA template. The sequences of the *Dirofilaria immitis* gene-specific primers used are detailed in Table 1. To detect human DNA in the nucleic acid isolate, a DNA oligo pair specific for an intergenic region of the *Homo sapiens* genome was utilized as a positive control [11]. The cycling conditions were as follows: 15 min at 95 °C for initial denaturation, followed by 40 cycles of amplification (30 s at 95 °C, 60 s at 60 °C, 20 s at 72 °C), and final extension for 7 min at 72 °C. PCR products were analyzed by agarose gel electrophoresis, incorporating a 50 bp DNA ladder (Thermo Fisher Scientific) as a size reference.

**Table 1:** Primer sequences applied in this study (COX1: Cyclooxygenase 1; RNA: Ribonucleic acid)

Gene	Forward primer (5’-3’)	Reverse primer (5’-3’)
*Dirofilaria immitis COX1*	GGACTTCTGTTTGGGGTCATCA	TCTTAACAGCCCTTGGAATAGCA
*Dirofilaria immitis* 18 S RNA	GGGACAAGCGGTGTTTAGC	GCACGCTGATTCCTCCAGT
Human intergenic region (10)	TGGAACTTCTGGAAGACACTG	TACACCACTCAAGGGAAACTG

## Results

### Clinical characteristics

Our case series encompasses all HPD cases identified between 2012 and 2023. Table 2 provides a summary of the clinical data obtained from the examined patients. Out of the 5 patients included, 3 were male. The mean age of the patients was 51 years (range: 37–62). HPD lesions were consistently found in the right lung, with 4 cases located in the lower lobe. Symptoms varied widely, ranging from incidental discovery during employment or routine medical examinations to presenting with fever and coughing. In all cases, imaging techniques revealed peripheral, tumor-like lesions, prompting surgical intervention. Lobectomy was performed in 2 cases, while video-assisted thoracic surgery (VATS) wedge resection was carried out in the remaining 3 cases. With regard to predisposing factors, only *patient 1* was known to reside in a rural area of Hungary. *Patient 3* was the sole individual clinically presenting with eosinophilia.

**Table 2:** Clinical features of the patients investigated (M: Male; F: Female; VATS: Video-assisted thoracic surgery)

Patient ID	Age (years)	Sex	Localization	Clinical presentation	Type of surgical treatment	Social factors
**1**	59	M	Right lower lobe	Frequent dry cough and occasional right-sided chest pain	Lobectomy	Lives in rural area
**2**	62	M	Right lower lobe	No symptoms; disease recognized incidentally during routine employment examination	Lobectomy	No data
**3**	45	M	Right lower lobe	Allergic symptoms for several years	VATS wedge resection	No data
**4**	37	F	Right upper lobe	Fever, dry cough	VATS wedge resection	No data
**5**	52	F	Right lower lobe	No symptoms; disease recognized incidentally during routine dispensary examination	VATS wedge resection	No data

### Pathological characteristics

**Fig. 1:**
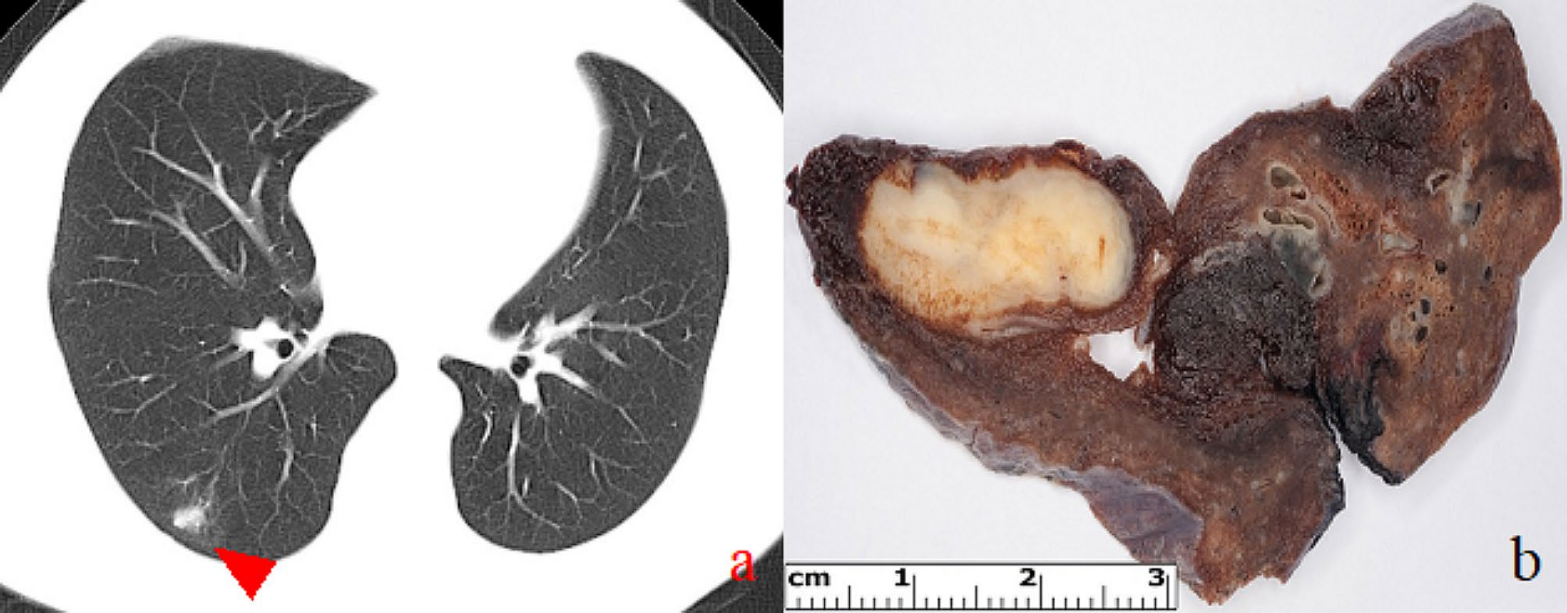
Radiological and macroscopic appearance of human pulmonary dirofilariasis. (**a**) On the chest CT scan, a peripheral coin lesion was observed in the lung parenchyma (red arrowhead). Otherwise, the CT scan found no abnormalities in the thorax. (**b**) Grossly, a well-defined, yellow-white nodule was situated in the subpleural area. Additionally, focal bleedings were present in the lung parenchyma

**Fig. 2:**
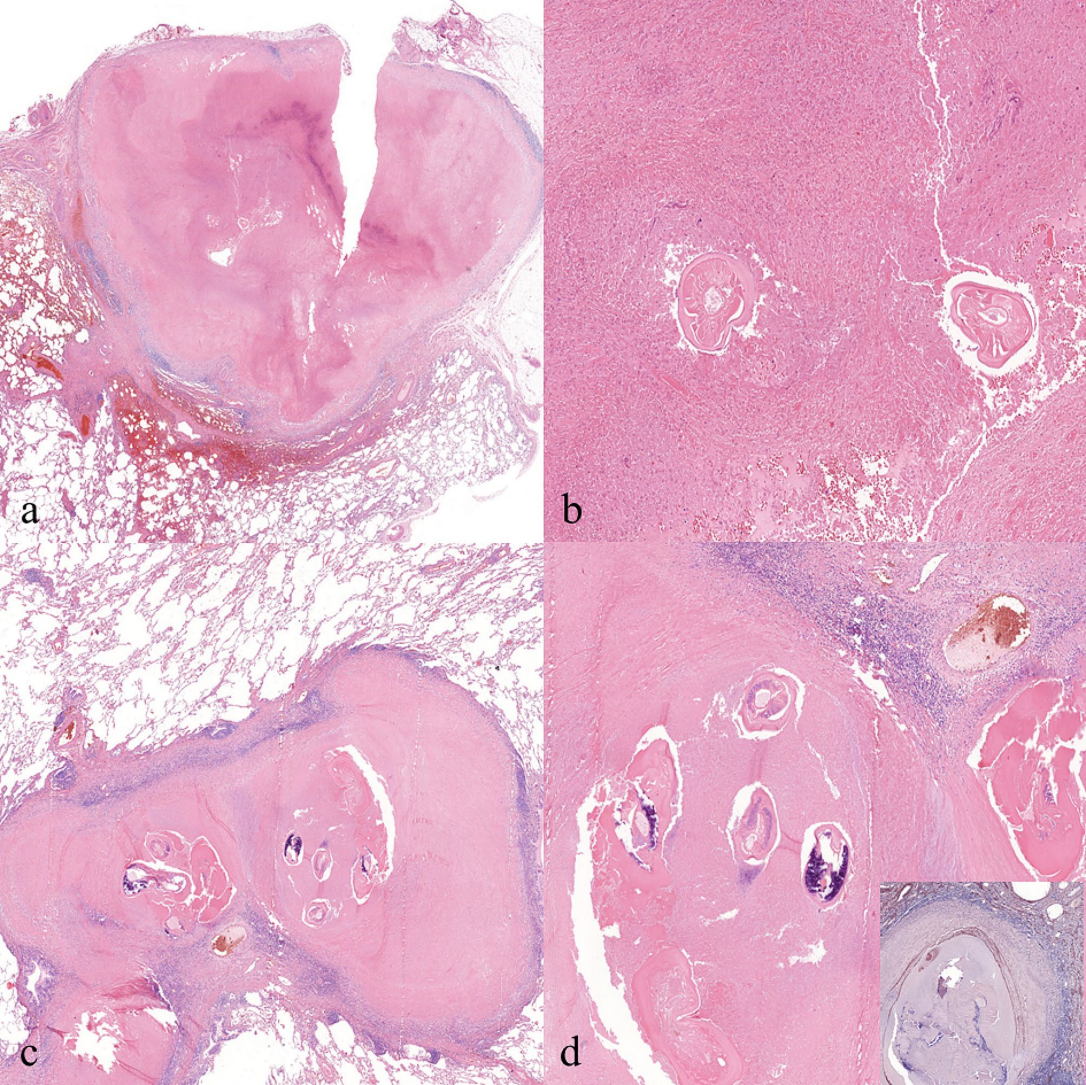
Representative pictures of the histological findings. (**a**) and (**b**) depict findings from *patient 4*. Notably, this case lacked an identifiable vessel wall. The necrotic lesion extended to the serosal surface, leading to fibrotic adhesion between the layers of the pleura. Scar tissue formation resulted in tissue tearing during the surgical procedure. (**c**) and (**d**) showcase observations from *patient 5*. This case exhibited the highest worm count, with some worms beginning to undergo dystrophic calcification. All samples were stained with hematoxylin and eosin. Images (**a**) and (**c**) were captured at a magnification factor of 50x, while images (**b**) and (**d**) were captured at a magnification factor of 100x. Additionally, an insert photo highlights the elastic layer of the pulmonary artery, stained with orcein and magnified at 50x

**Fig. 3:**
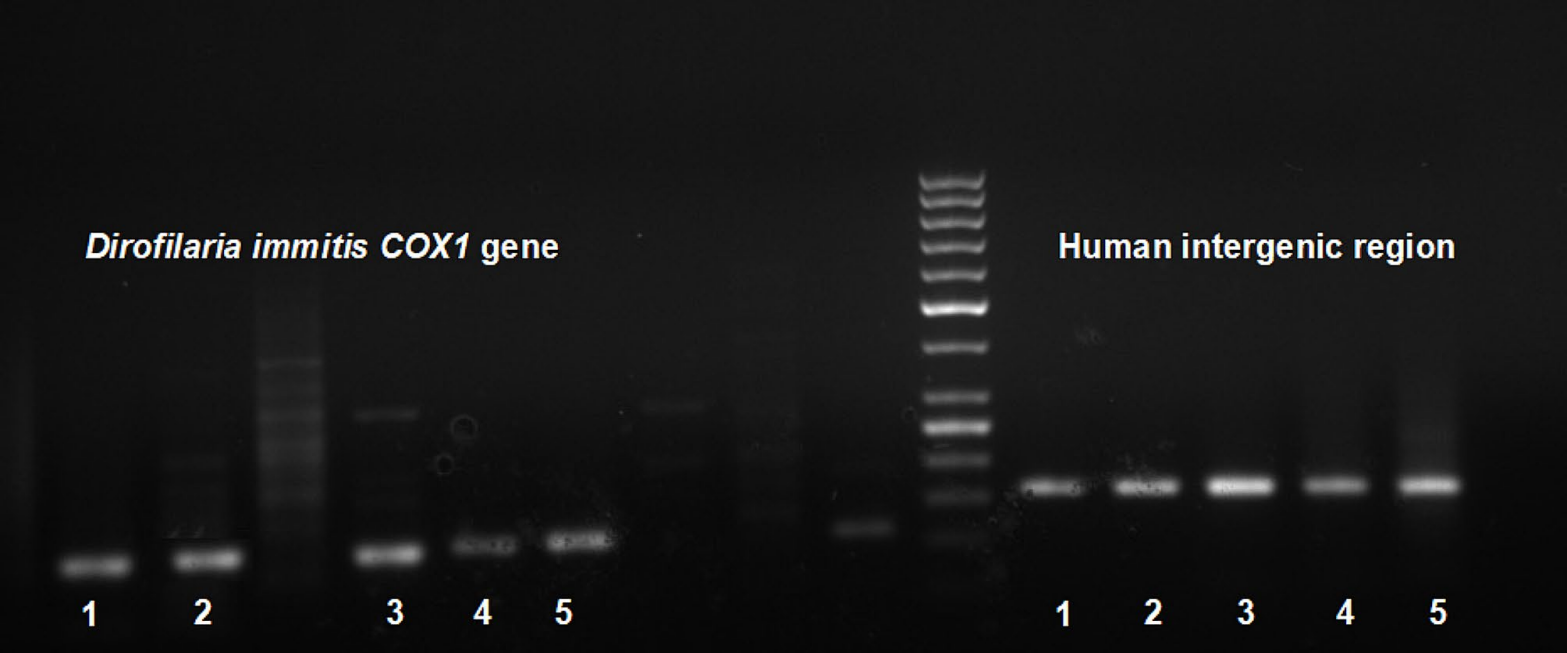
Amplicons of the polymerase chain reaction carried out. We were able to amplify the *COX1* gene of the *Dirofilaria immitis*, and the intergenic region of the human DNA in each case. The former confirmed the species of the helminth, and the latter proved that the host was human. COX1: Cyclooxygenase 1; DNA: Deoxyribonucleic acid

Table 3 sums up the morphological features of the examined cases. The lesions exhibited an average size of 16.2 mm in greatest dimension and were consistently characterized as subpleural and well-circumscribed, appearing either yellow or white (Fig. 1). Histologically, a necrotizing granulomatous reaction was uniformly observed across cases, with central remnants of helminths notably present within thrombosed arteries in *patients 1*, *2*, *3*, and *5* (Fig. 2). Surrounding tissue displayed predominantly foreign body type reactions, chronic inflammation, and fibrosis, while ovules were not detected in any case. To rule out tuberculosis and fungal infections, Ziehl-Neelsen and periodic acid-Schiff (PAS) staining were performed in all cases. Grocott staining was additionally conducted in *patients 2* and *5*, with *Mycobacterium tuberculosis* immunohistochemistry (carried out specifically in *patient 5*). PCR analysis conclusively established the diagnosis of HPD in all cases (Fig. 3).

**Table 3:** Morphological features of the human pulmonary dirofilariasis cases analyzed (PAS: Periodic Acid-Schiff). *For the immunohistochemistry, rabbit polyclonal antibody (ab905, Abcam) was applied

Patient ID	Size (mm)	Grossing results	Histological features	Examination carried out to identify infectious origin	PCR result
**1**	15 × 9 × 4	Subpleural, well-circumscribed, yellow lesion	Necrotizing granulomatous reaction with thick, fibrotic capsule with central thrombosed pulmonary artery, containing helminth remnant. Lymphocytes, histiocytes, Langhans and foreign body-type giant cells in the surrounding areas.	Ziehl-Neelsen, PAS	*Dirofilaria immitis*
**2**	21 × 15 × 15	Subpleural, well-circumscribed, white lesion	Necrotizing granulomatous reaction with central helminth remnant. Foreign body type reaction, chronic inflammation, fibrosis.	Ziehl-Neelsen, PAS, Grocott	*Dirofilaria immitis*
**3**	6 × 5 × 5	Subpleural, well-circumscribed, yellow lesion	Necrotizing granulomatous reaction with central helminth remnant. Foreign body type reaction, chronic inflammation, fibrosis.	Ziehl-Neelsen, PAS	*Dirofilaria immitis*
**4**	21 × 14 × 7	Subpleural, well-circumscribed, white lesion	Necrotizing granulomatous reaction with central helminth remnant. Foreign body type reaction, calcification and granulation tissue formation.	Ziehl-Neelsen, PAS	*Dirofilaria immitis*
**5**	18 × 11 × 6	Subpleural, well-circumscribed, white lesion	Necrotizing granulomatous reaction with central helminth remnant. Foreign body type reaction, chronic inflammation, fibrosis.	Ziehl-Neelsen, PAS, Grocott, Mycobacterium tuberculosis* immunohistochemistry	*Dirofilaria immitis*

## Discussion

Dirofilariasis, a prevalent parasitic infection among canines in warm climate regions, primarily involves *Dirofilaria repens* and *Dirofilaria immitis* within Europe [12]. In locations such as the Po River Valley, canine infection rates may range from 50 to 80% [13]. Similarly, Serbia and Romania report substantial rates of canine infections, at 22.01% and 23.07%, respectively [14]. The occurrence of autochthonous *Dirofilaria immitis* infection in dogs in Hungary was first documented in 2002 [15]. While sporadic cases of Dirofilaria infection, mainly affecting the ocular and subcutaneous regions, have been reported previously in Hungary, instances of human pulmonary dirofilariasis have yet to be documented in the literature [16–20]. Interestingly, subcutaneous infections, involving mainly the head and neck region are linked to *Dirofilaria repens*, while HPD is primarily attributed to *Dirofilaria immitis* infection [21]. Although HPD remains relatively uncommon in Europe, recent reports from Mediterranean countries such as Italy, Greece, and Spain have been published [22]. The first PCR-confirmed HPD case from Italy was reported in 2022 [21]. It is essential to note the absence of prospective analyses on human Dirofilaria infections, thus the exact incidence of HPD remains unknown. Available data rely on single case reports or retrospective analyses of case series. For instance, in 1997, Raccurt reported 71 human dirofilariasis cases, primarily subcutaneous, with only 2 pulmonary manifestations. Based on these findings, he estimated France to have the second-highest human incidence of dirofilarisis in Europe [23]. Similarly, in 2001, Pampiglione investigated 60 new Italian human Dirofilaria cases, of which only 2 involved the lungs [24]. In non-European regions, Atsumi et al. reported 13 HPD cases from Okinawa, observing the highest occurrence rate in Japan [25]. In the USA, 116 HPD cases have been reported, predominantly in the southeast regions [26]. In temperate regions like Hungary, human infections most likely result from travel to endemic areas, immigration, and climate change [27]. Clinically, HPD should be considered in the differential diagnosis of subpleural coin lesions in the lower pulmonary lobes. Key risk factors include advanced age, canine ownership, and outdoor activities predisposing individuals to mosquito bites [2, 28–31].

Macroscopically, HPD presents as a homogeneous round nodule, while histologically, the helminth exhibits a distinctive thick cuticle with 3 discernible layers, featuring internal longitudinal ridges and well-developed reproductive organs and intestines. These morphological features are characteristic for Dirofilaria species. In addition, the worm is typically located within thrombosed pulmonary arteries and surrounded by hemorrhagic infarction along with granulomatous infiltration of neutrophils, eosinophils, plasma cells, and giant cells. Calcification of the parasite is infrequently observed, and the presence of the helminth is crucial for definitive diagnosis, necessitating processing of the entire nodule [32–34].

The primary histological differential diagnoses for HPD include either granulomatous lung disorders such as tuberculosis, granulomatosis with polyangiitis, polyarteritis nodosa, or pulmonary thromboembolism. Distinguishing Dirofilaria species from other worms is typically straightforward, with *Enterobius vermicularis* lacking fully developed reproductive organs and Dirofilaria residing solely within pulmonary vessels [32]. PCR, a highly sensitive method, can detect the specific ribonucleic acid of the parasite even at low concentrations, corroborating routine histological diagnosis.

Clinically, HPD is typically detected incidentally during CT scans, conducted for unrelated reasons. Clinical symptoms seldom manifest and are nonspecific. Given HPD’s radiological resemblance to primary or metastatic lung cancer, surgical resection is often pursued. As discussed earlier, histological diagnosis is usually straightforward. Detection of living microfilariae in the blood is exceedingly rare, and in the majority of cases, surgical removal is curative, without necessitating systemic anti-parasitic therapy [32]. As limitation of our study, we have to mention two factors. Firstly, this is a retrospective analysis of the histologically diagnosed cases, therefore we are not able to estimate the incidence of HPD incidence accurately. From Central Europe, solely case reports exist, therefore, it is impossible to directly compare our data with that of other temperate climate countries. Consequently, we present our findings as a reference point for future studies to build upon. And secondly, we had limited access to the clinical data, hence our study lacks some relevant parameters including owning domestic animals and traveling data.

## Conclusions

The incidence of HPD in Europe is currently uncertain and in need of further studies, as only a limited number of cases have previously been reported. Due to the confluence of factors including immigration, burgeoning global tourism, and the intricate impacts of climate change, a projected escalation in the incidence of HPD across Europe may be expected. To address this concern, we present the inaugural Hungarian case series, encompassing 5 instances of potentially human autochthonous *Dirofilaria immitis* infection. This comprehensive cohort spans all documented cases over the past 12 years, all of whom underwent surgical intervention at the distinguished University of Szeged. Our study underscores the imperative consideration of HPD in cases presenting with stable subpleural coin lesions of uncertain etiology in temperate regions. Diagnosis relies heavily on meticulous histological examination of the lesion, followed by confirmation through molecular analysis. While a preoperative biopsy may provide some diagnostic insight, its efficacy is contingent upon the presence of identifiable parasite fragments. However, achieving a correct diagnosis without complete lesion excision poses a considerable challenge.

## Data Availability

All data generated or analyzed during this study are included in this article. Further inquiries can be directed to the corresponding author.

## Abbreviations

COX1: Cyclooxygenase 1
CT: Computed tomography
DNA: Deoxyribonucleic acid
HPD: Human pulmonary dirofilariasis
PAS: Periodic acid-Schiff
PCR: Polymerase chain reaction
RNA: Ribonucleic acid
VATS: Video-assisted thoracic surgery
